# Investigating the Devil’s tree(*Alstonia scholaris*) for its health-safeguardingproperty and antipathogenic activities against fungi and helminthparasites of veterinary importance

**DOI:** 10.5455/javar.2025.l993

**Published:** 2025-12-25

**Authors:** Lal Ngaihmanawmi, Pawi Bawitlung Lalthanpuii, Lalbiakngheti Tlau, Lucy Lalawmpuii, Lal Rosangpuii, Lal Nundanga, Kholhring Lalchhandama

**Affiliations:** 1 Department of Forestry, Mizoram University, Aizawl, India; 2 Department of Botany, Government Kolasib College, Kolasib, India; 3 Department of Life Sciences, Pachhunga University College, Mizoram University, Aizawl, India; 4 Department of Biochemistry, Government Zirtiri Residential Science College, Aizawl, India

**Keywords:** Antifungal, antioxidant, antiparasitic, phytocompound, tapeworm

## Abstract

**Objective:**

The study was formulated to analyze the health benefits and antifungal and anthelmintic properties of Alstonia scholaris, specifically its bark, as claimed in Mizo traditional medicine. Biochemical assays were set up to evaluate the antioxidant potential and bioactivity tests on activity against pathogenic fungi and helminth parasites.

**Materials and Methods:**

Alstonia scholaris bark extract was prepared in petroleum ether, a non-polar solvent. Phytochemical detections were performed for 11 chemical tests. The total antioxidant, flavonoid, and phenol contents were determined. The free radical-scavenging reactions were determined using 2,2-diphenyl-1-picrylhydrazine (DPPH) scavenging and ferric ion-reducing antioxidant power (FRAP) assays. The antifungal activity was tested by an agar diffusion method. Anthelmintic activity was assessed by survival assay, scanning electron microscopy, and histology.

**Results:**

Phytosterols were identified as the main bioactive compounds. The total antioxidant was estimated at 6.4305 mg ascorbate equivalent per gram, flavonoid at 128.03 mg gallic acid equivalent per gram, and phenol at 10.72 mg pentahydroxyflavone equivalent per gram. The FRAP assay demonstrated a concentration-dependent scavenging of ferric (Fe⁺³) cations. The DPPH scavenging reaction showed the half-maximal inhibitory concentration at 136 µg/ml. It was found that the plant extract was effective against Candida albicans, Neocosmospora keratoplastica, and Neosartorya fumigata, with the highest degree of inhibition against C. albicans. It exhibited activity against the poultry tapeworm, Raillietina echinobothrida. Light and electron microscopy revealed signature antiparasitic effects on different parts of the parasite body

**Conclusion:**

The study vindicated the medicinal properties of A. scholaris in terms of antioxidants, antifungal, and antiparasitic activities.

## Introduction

Despite the proclaimed successes in the development of antimicrobial and anthelmintic medications in the 20th century, which saved millions of lives from debilitating diseases, the clinical management of microbial and helminth infections remains in a dire situation. The major factor is the evolution and rapid propagation of drug resistance, progressing to multidrug resistance (MDR) and extensively drug resistance (XDR), which has created the superbugs that are virtually invincible to all the drugs available for their elimination [1,2]. The phenomena of MDR and XDR turned into a global health crisis, and the dangers posed by the pathogens are as critical as they were in the pre-antibiotic era. Millions of deaths, ranging from over 1to 5 million, were attributed to antibiotic resistance alone in recent years [3].

Pathogens, once thought to be well contained with proper therapeutic management, became more virulent and deadly than before. For instance, *Mycobacterium tuberculosis* and *Helicobacter pylori* infections are increasing steadily and lead to escalation of mortalities due to tuberculosis and gastric cancer [4,5]. Antiparasitic resistance in major helminth parasites is the primary cause of substantial economic losses in the animal industry, and the solution does not appear to lie within our current system of parasite management [6]. Lessons from the veterinary cases and reports of diminished efficacy of antiparasitic drugs used for human infections pose a greater concern, as helminthiasis is emerging as the most prevalent infectious disease in humans [7,8].

Natural products are a major source of pharmaceutical drugs, and many medications in clinical use are derived from plants or synthesized chemically from them [9,10]. Medications obtained from medicinal plants have been triumphantly used in the treatment of the most serious to the mildest diseases. Camptothecin from *Camptotheca acuminata*, paclitaxel from *Taxus brevifolia*, and vinca alkaloids (vinblastine and vincristine) from *Catharanthus roseus* (including their synthetic derivatives, vindesine, vinflunine, and vinorelbine) are the frontline medications in cancer therapy [11,12]. Quinine from *Cinchona pube* *scens* and artemisinin from *Artemisia annua*, with their chemical derivatives, serve as the primary treatments of malaria and other parasitic infections [13]. Cocaine from *Erythroxylum coca*, digitoxin from *Digitalis* species, pilocarpine from *Pilocarpus microphyllus*, and codeine from *Papaver so* *mniferu* *m* are becoming household names in medicine for various medical conditions [14]. Thus, it is essential to conduct further explorations of well-established medicinal compounds to identify novel lead molecules for various infections.

An interesting plant is *Alstonia scholaris* (L.) R.Br., a deciduous evergreen tree naturally growing in Africa, Asia, and Australia [15]. In Asian cultures, such as those in China, India, and Malaysia, it is a well-established therapy for blood disorders, particularly in *Plasmodium* infections and severe fevers [16]. Based on applications in traditional Chinese medicine, several indole alkaloids isolated from the plant are undergoing clinical trials for the treatment of respiratory diseases [17]. One such compound, echitamine, has been actively experimented with as a lead compound for an antimalarial drug [18]. The plant is also one of the best-documented species in India, with recorded applications as an analgesic (specifically for rheumatoid arthritis), anticancer, antiparasitic, anti-asthmatic, anti-inflammatory, antimicrobial (especially in leprosy and jaundice), and laxative agent [19–24]. It is given an unflattering common name, the Devil’s tree, due to its massive production of pollen that causes acute allergies during the flowering season [25]. It bears a characteristic arrangement of leaves that arise from a single node, and geographical varieties are recognized from the number of leaf whorls in each node, which can be from a few to many. In mainland India, the tree is known for its typical seven-leafed whorls [23,24].

Mizoram is the farthest northeastern state in India, situated within the Indo-Burma biodiversity hotspot, bordered by Bangladesh to the west and Myanmar to the east. The scholaris in Mizoram identified so far are distinctively characterized by eight-leafed whorls, which is the basis for the Mizo vernacular name ṭhuamriat (literally, “eight whorled/branched”). According to Mizo folk medicine, the leaves and bark are effective remedies for asthma, diarrhea, dysentery, ear infections, heart diseases, hypertension, malaria, snake bites, antivenom, and typhoid fever [26–29]. The medicinal properties of its leaves had been reported [15,24], but there were no reports on the specific antifungal and anthelmintic properties. Especially bark, as a source of therapeutic agents, as in traditional tribal medicine, remains unexplored. It is therefore crucial to have a scientifically sound understanding of the acclaimed medicinal uses of the bark extract. The experimental study was thus organized to analyze the biochemical constituents, antioxidant status, and antipathogenic activities against pathogenic fungi and intestinal helminths of veterinary importance. The overall findings will provide a better understanding of the fundamental pharmacological properties of *A. scholaris* growing in Mizoram.

## Materials and Methods

### Ethical statement

All experimental procedures involving organisms were conducted under the approval of the Institutional Animal Ethics Committee of Pachhunga University College (PUC-IAEC-2022-02). The approval was issued on 15 March 2022.

### Plant source

The different parts of *A. scholaris* were obtained from the plantation at Lungdai, Mizoram, Northeast India, which is situated at 23°47'57.01"N 92°47'33.75"E. The flowers and leaves were made in herbaria and identified (vide BSI/ERC file number 102-17-05-23) at the Botanical Survey of India (Shillong Centre), Meghalaya, Northeast India. Preserved herbaria bearing the catalogue code PUC-AS-22-01 are maintained.

### Extract preparation

The barks of *A. scholaris* were peeled off and cleansed in dechlorinated water. After pulverizing the material into small pieces with a grinder, it was dried in an ambient environment for 1 month. The dried sample in batches of 350 gm was loaded into a Soxhlet apparatus of 5-L capacity. Hot extraction was performed using petroleum ether, an extremely nonpolar solvent with a polarity index of 0.1. Complete extraction was accomplished in 4 days. The solvent was removed and recycled by vacuum-pressurized evaporation in Rotavapor^®^ R-100 (Buchi, Flawil, Switzerland) to produce a concentrated extract.

### Qualitative phytochemical detection

Secondary metabolites present in *A. scholaris* bark extract were detected using standard pharmacognostic chemical tests [30]. 11 chemical group tests were performed including Dragendorff’s test, Mayer’s test, picric acid test and Wagner’s test for alkaloids; ammonia water hydroxide test and Borntrager’s test for anthracenediones; Barfoed’s test, Benedict’s test, iodine test, Fehling’s test and Molisch’s test for carbohydrates; lead acetate test, Shinoda reaction and Clemmensen reduction for flavonoids; Keller-Kiliani test, Legal’s test and Liebermann’s test for glycosomes; ethanol test for gums; Liebermann-Burchard’s test and Salkowski reaction for sterols; biuret reaction and ninhydrin test for amino acids and proteins; Benedict’s test and Molisch’s test for reducing sugars; froth test for saponins; iron(III) chloride reaction, lead acetate reaction and dichromic acid test for tannins.

### Flavonoid content

Aluminium reduction reaction was performed to estimate the number of total flavonoids in *A. scholaris* extract [31]. Firstly, a stock solution of 100 mg/ml of the extract was prepared. 3,3’,4’,5,7-Pentahydroxyflavone was employed as a reference compound and made in concentrations of 10, 20, 40, 60, 80, and 100 µg/ml. 2 ml of dechlorinated water was added to each sample. Plant extract solution was made from 1 ml of the stock sample and 2 ml of water. After mixing for 5 min, they were added with 300 µl each of aluminum trichloride (made at 10%) and sodium nitrite (at 5%). After 6 min, 2 ml of caustic soda was mixed with all the samples. Each sample was made to a total of 10 ml by adding dechlorinated water. They were allowed to remain for 60 min. The absorbance was taken at the wavelength of 510 nm in a double-beam LT39 UV-vis photometer (Labtronics, India). The linear regression of the reference compound was plotted, from which the amount of flavonoid was calculated as milligrams of pentahydroxyflavone equivalent per gram (mg PFE/gm) of the dried extract.

### Antioxidant content

The total antioxidant constituent was quantified using the phosphomolybdic acid assay [32]. A reference antioxidant, ascorbate (L-ascorbic acid), was made into six concentrations as in the total antioxidant assay. A reagent mixture was prepared with 4 mM ammonium paramolybdate, 28 mM sodium orthophosphate, and 0.6 M hydrogen sulfate. 3 ml of the reagent was mixed with 100 µl of each of the ascorbate samples and the plant extract. The mixtures were maintained at 95°C in a chemical incubator for a stable reaction. After 1.5 h, the solutions were taken out in an ambient environment and left to cool. The absorbance was read at 695 nm. From the standard graph plotted for ascorbate, the antioxidant content was calculated as milligrams of ascorbate equivalent per gram (mg ABE/gm) of the dried extract. Each test was executed in triplicate.

### Phenolic content

The phenolic content was quantified from the phosphomolybdic acid and phosphotungstate reaction [33]. Gallic acid, used as a standard reference, was prepared in increasing concentrations as in previous assays. Five milliliters of Folin-Ciocalteu reagent was mixed with each sample and left for 3 min to allow for complete chemical reaction in an ambient environment. The reagent was also mixed with 200 µl of the plant extract solution. 4 ml of 0.7 M disodium carbonate was added to all samples and whisked using a magnetic stirrer for 60 min. The absorbance was recorded at 765 nm and adjusted against a blank solution prepared from Folin-Ciocalteu reagent, methanol, and disodium carbonate in a 5:1:4 ratio. The total phenol concentration was determined using the calibration graph of gallic acid and calculated as milligrams of gallic acid equivalent per gram (mg GAE/gm) of the dried extract.

### Ferric ion-reducing antioxidant power (FRAP) assay

The antioxidation capacity against ferric ion was evaluated by the potassium ferricyanide reaction [34]. Ascorbate was taken as a reference antioxidant. Ascorbate and *A. sc* *holari* *s* extract were prepared in increasing concentrations as in previous assays. 10% Potassium hexacyanoferrate (III) and phosphate buffer (pH 6.6) were added to 1 ml of all the samples. After centrifugation at 3,000 rpm for 10 min, from each sample, the supernatants (2.5 ml each) were taken and mixed with 2.5 ml of dechlorinated water. 0.1% Iron (III) chloride was added to all samples to make a 3 ml volume. For a reference blank reading, a mixture of 2.5 ml of potassium ferricyanide, 1 ml of dechlorinated water, and 2.5 ml of phosphate buffer was used. The absorbance was read at 700 nm against the blank solution.

### 2,2-diphenyl-1-picrylhydrazine (DPPH)-scavenging activity assay

The capacity to scavenge cellular oxidants was assessed by the DPPH degradation method [35]. Both A. scholaris extract and dibutylhydroxytoluene, a standard antioxidant, were made in different concentrations as in the previous. To 3 ml of all the samples, 500 µl of 1 mM DPPH was added. Negative control consisted of DPPH and methyl alcohol in a 1:3 ratio. All the samples underwent reaction at 37°C for 30 min. The intensity or optical density (OD) was read at 517 nm. The degree of oxidant-scavenging action was estimated as:


$$\mathrm{Scavenging}\;\mathrm{action}\mathrm{(\%)}=\frac{\mathrm{Control}\;\mathrm{OD}-\mathrm{Extract}\;\mathrm{OD}}{\mathrm{Control}\;\mathrm{OD}}\times 100$$

half-maximal inhibiting concentration (IC50) was calculated from a dose-response curve prepared from log10 at 1, 0.5, and 0.25 mg/ml.

### Fungal inhibitory assay

The fungal inhibitory property of *A. scholaris* extract was evaluated using agar diffusion based on the poisoned food technique [36], one of the most sensitive tests for antifungal activity [37]. *Candida albicans* (ATCC 26790), *Neosartorya fumigata* (ATCC 204305), and *Neocosmospora keratoplastica* (ATCC 36031) were obtained from the HiMedia company, Mumbai, Maharashtra, India. Each fungal specimen was applied to a sterilized growth medium consisting of potato-dextrose agar kept in Petri dishes. The cultures were maintained at 27°C ± 2°C and allowed to proliferate for 7 days in a sterile chamber (Igene IG-95I, New Delhi, India). The plant extract was prepared in 1.25, 2.5, 5, and 10 mg/ml using 20 ml of fresh and liquefied potato-dextrose agar. A negative control was set aside, having only the agar medium. 6 mm discs were cut from the fully grown fungi using a disinfected cork borer and were inoculated at the center of the Petri dishes in which culture media were maintained. All the culture dishes were sealed airtight with sterile parafilm. While incubating at 27°C ± 2°C for 7 days, the growth zones were recorded every day. The total inhibition of growth was estimated in percentage against the growth zones of the control.

### Antiparasitic susceptibility assay

*Raillietina echinobothrida* Megnin, 1,880 were obtained by dissection of the intestines of chickens, *Gallus domesticus* Linnaeus, 1,758. Following the standardized helminth survival assay [38], the tapeworms were maintained in a microbiological incubator kept at 37°C± 1°C in culture media made of 0.9% phosphate-buffered saline (PBS) and 1% sulfinylbismethane (SBM). They were exposed to *A. scholaris* bark extract at 5, 10, and 20 mg/ml made with the culture media. Negative control consisted of tapeworms maintained in only PBS + SBM, and positive control was treated with a reference antiparasitic drug, albendazole. For each test, three tapeworms were used, and each test was conducted in triplicate. Survival value was given in mean ± standard deviation.

### Histology and light microscopy

*Alstonia scholaris* extract-treated *R. echinobothrida* were processed for histology to determine possible alterations in the structure of the anatomical parts. The worms were cleansed in PBS solution and then immersed in Bouin fluid overnight for complete tissue fixation. The fixative was washed off in distilled water, and the tissues were dehydrated from 30% to 100% ethyl alcohol. They were dealcoholized using xylene and then made into cubes using paraffin. Sections were cut at ~5 µm thickness in an MRM -ST microtome (Medimeas, Haryana, India). The tissue sections were spread on glass slides and dehydrated in increasing concentrations of ethanol. After double staining with hydroxybrazilin and bromo acid, they were fixed on slides and visualized under an Eclipse CiE DSRi2 microscopic analyzer (Nikon, Tokyo, Japan).

### Scanning electron microscopy

The anthelmintic effect on the tapeworm was assessed using scanning electron microscopy, as the technique allows identification of the finest details of structural damages [38]. *Alstonia* *scholaris* extract-treated *R. echinobothrida* was washed thoroughly in PBS. They were cut into small sections and kept in methanol (10% buffered with PBS at pH 7) at 4°C for tissue fixation. After 4 h, complete dehydration was performed through ascending grades of acetone. To stabilize the tissues, the tapeworms were immersed in tetramethylsilicane for 10 min. The solvent was removed by evaporation in an air-dryer containment at 25°C. The tissue was smeared with gold using a Hitachi MC1000 coater, and the micrographs were generated from a Hitachi TM4000Plus II electron microscope (Tokyo, Japan).

### Statistical analysis

Comparison of the different experimental groups was carried out with analysis of variance and Tukey’s group difference test. Significance level between groups was taken at *p* < 0.05. Data analysis and generation of graphs were done in Prism 10.4.1 (GraphPad, Boston, USA).

## Results

### Phytocompounds

Based on standard chemical detection procedures, 11 phytocompounds were tested on the petroleum ether extract of *A. scholaris* bark. Alkaloids, anthracenediones, carbohydrates, flavonoids, glycosomes, gums, proteins and amino acids, reducing sugars, saponins, and tannins were not detected by the specific tests employed for each. Phytosterols appeared to be the main phytocompounds, as indicated by both the Liebermann-Burchard test and the Salkowski reaction (Table 1). This could be expected, as the Petroleum Ether is a highly nonpolar solvent and would most likely eliminate most of the polar molecules, such as alkaloids, anthracenediones, carbohydrates, and proteins, during hot extraction.

**Table 1.** Qualitative detection of compound from the chloroform extract of *A. scholaris* bark.

Phytocompounds	Name of test	Extract indication
Alkaloid	1. Dragendorff’s test	-
	2. Mayer’s test	-
	3. Picric acid test	-
	4. Wagner’s test	-
Anthracenedione	1. Ammonia water test	-
	2. Borntrager’s test	-
Carbohydrate	1. Barfoed’s test	-
	2. Benedict’s test	-
	3. Fehling’s test	-
	4. Molisch’s test	-
Flavonoid	1. Lead acetate test	-
	2. Shinoda reaction	-
	3. Clemmensen reduction	-
Glycosomes	1. Keller-Kiliani test	-
	2. Legal’s test	-
	3. Liebermann’s test	-
Gum	1. Ethanol test	-
Phytosterol	1. Liebermann-Burchard test	+
	2. Salkowski reaction	+
Protein and amino acid	1. Biuret reaction	-
	2. Ninhydrin test	-
Reducing agent (sugar)	1. Benedict’s test	-
	2. Molisch’s test	-
Saponin	1. Froth test	-
Tannin	1. Iron (III) chloride reaction	-
	2. Lead acetate reaction	-
	3. Dichromic acid test	-

+ = presence; - = absence.

### Flavonoid

**Figure 1.**
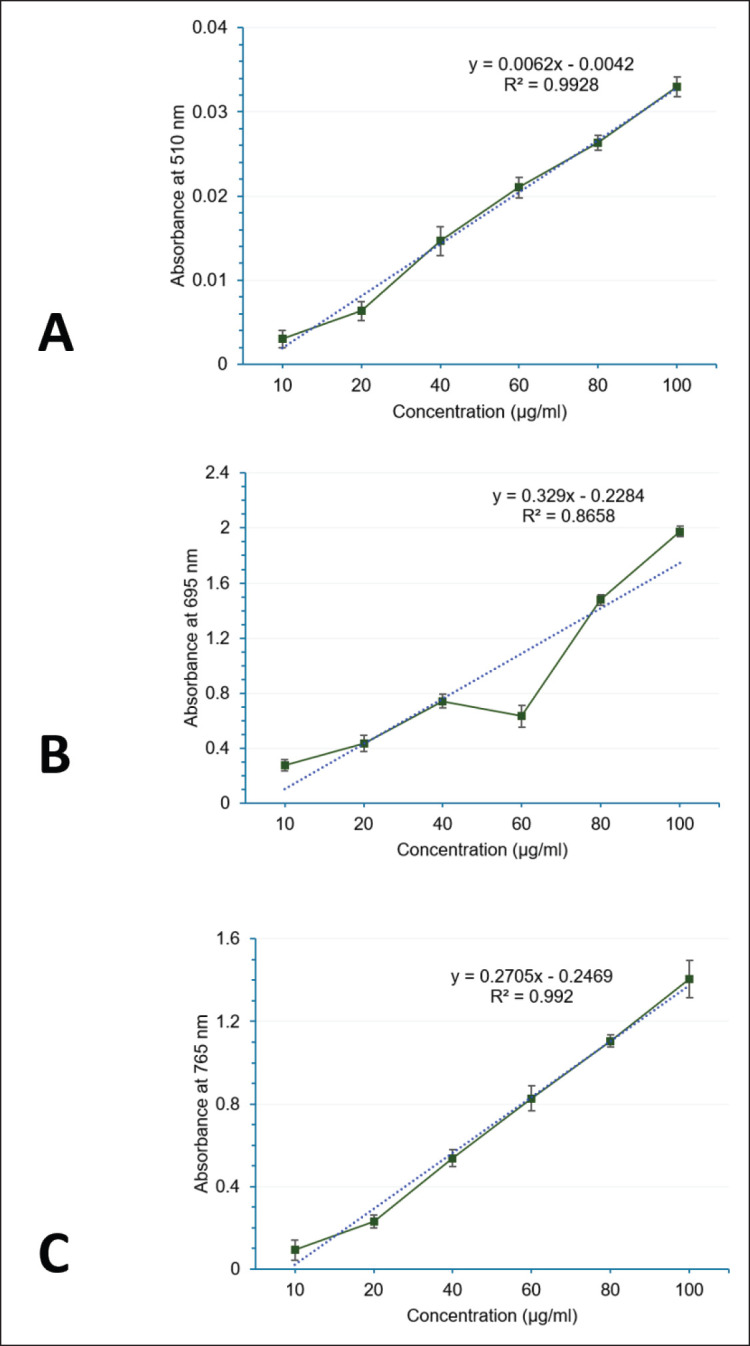
Standard graphs for quantifying antioxidant contents in *A. scholaris* bark extract. (A) Pentahydroxyflavone (quercetin) for the total flavonoid. (B) Ascorbate for the total antioxidants. (C) Gallic acid for the total phenol. Bars represent standard error of the Mean (*n* = 3).

The total flavonoid component of *A. scholaris* extract was calculated from the linear graph of pentahydroxyflavone as shown in Figure 1A. The calculation indicated that the plant contains a total flavonoid of 128.03 ± 5.96 mg PFE/gm of the dried extract.

### Total antioxidant

The linear graph of standard ascorbate for determining the total antioxidant content is illustrated in Figure 1B. From regression analysis, the total antioxidant level of the plant sample was estimated at 6.43 ± 0.05 mg ABE/gm of the dried extract.

### Phenol

The calibration curve of gallic acid as shown in Figure 1C was taken for the estimation of the total phenol present in the plant extract. Quantification from the linear graph gave the total phenol as 10.72 ± 0.13 mg GAE/gm of the dried extract.

### Antioxidant activity

**Figure 2.**
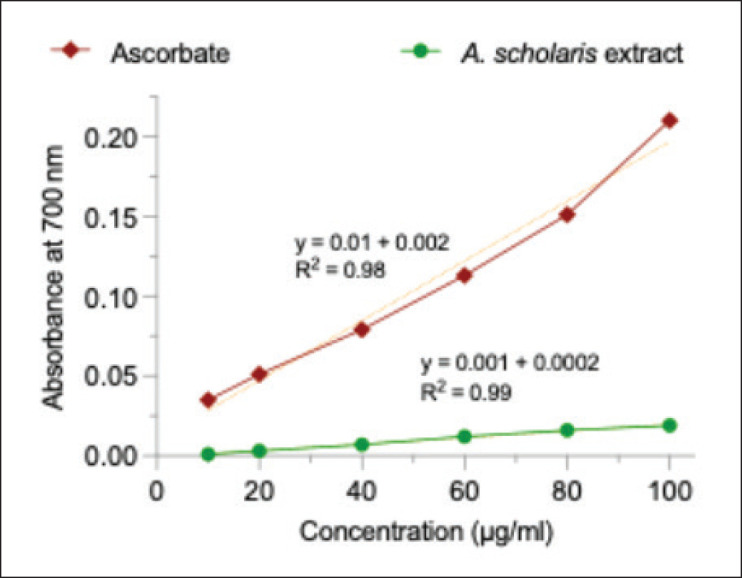
Ferric ion reducing activity of *A. scholaris* bark extract and ascorbate.

The FRAP assay indicated that *A. scholaris* bark extract and the reference compound, ascorbate, exhibited dose-dependent efficacy. The plant extract showed lower activity than ascorbate at all concentrations tested (Fig. 2). Ascorbate activity drastically increased at higher concentrations, while that of the plant extract showed a linear increase. The data shows that the plant extract has the chemical property to convert metabolically harmful ferric cations (Fe⁺³) to less innocuous ferrous cations (Fe⁺²).

**Figure 3.**
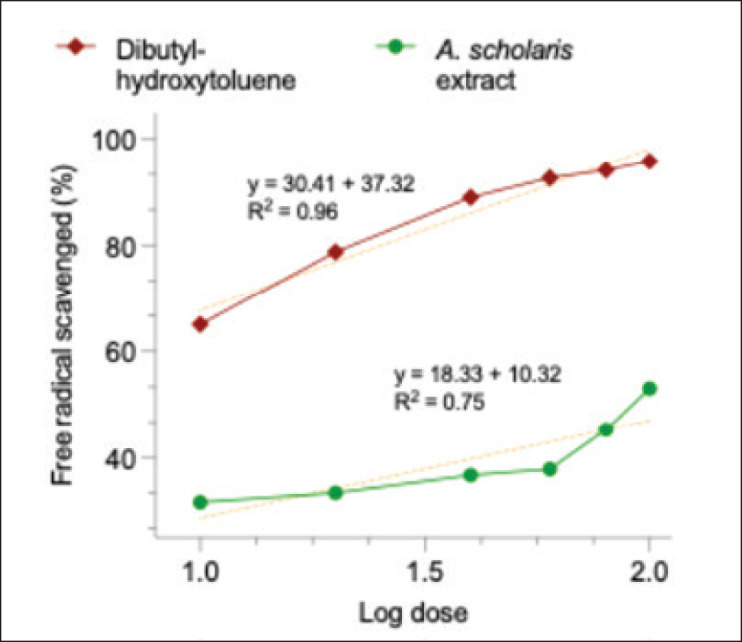
DPPH scavenging activity of *A. scholaris* bark extract and dibutylhydroxytoluene.

The antioxidant property evaluated from the chemical scavenging of DPPH free radical showed a distinct concentration-dependent reaction (Fig. 3). The standard antioxidant, dibutylhydroxytoluene, also showed similar activity with higher efficacy. From the log dose calculation, the plant extract had an IC50 of 136 µg/ml, which was far less than that of dibutylhydroxytoluene at 5.60 µg/ml.

### Fungal inhibition

**Figure 4.**
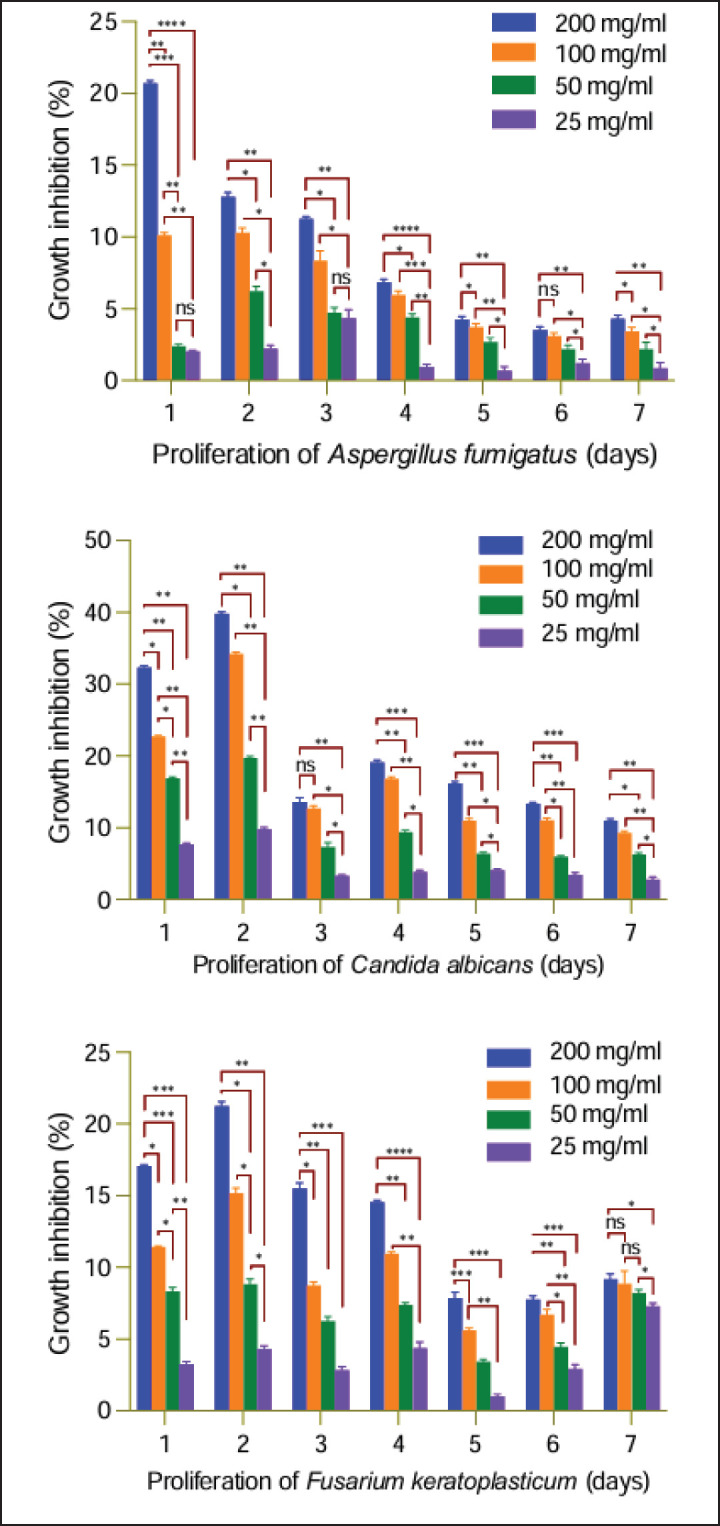
Comparison of the antifungal action of *A* *. scholaris* bark extract against three fungal species. Values are in means ± standard error of means (*n* = 3); ****p < 0.0001, ***p < 0.002, **p < 0.001, *p < 0.05, and ns = not significant.

The fungal inhibition of *A. scholaris* extract upon *C. albicans*, N. fumigata, and *N. keratoplastica* is presented in Table 2. Under normal conditions, the three species proliferated uniformly during 1-week culture characterized by enlarged circles of growth areas around each sample. In control experiments, maximal growth was observed among all experimental cultures for all fungi. Slowest growth was noted for *C. albicans*, followed by *N. keratoplastica*, while *N. fumigata* proliferated the fastest. The plant extract effectively inhibited progressive proliferation against the three fungi. However, insignificant inhibitions were seen in the minimum concentration (25 mg/ml) in all the fungi, compared to the control. Inhibition was slowest in *N. keratoplastica*, while the statistically highest inhibitions were seen in *C. albicans*. The total inhibition against different fungi at the highest concentrations was 4.3% against *N. fumigata*, 10.9% against *C. albicans*, and 9.2% against *N. keratoplastica*. Statistical comparisons of the degree of proliferation among the three fungi under various conditions are depicted in Figure 4.

**Table 2.** Effects of *A. scholaris* bark extract on the growth pattern of fungi.

Duration	Growth zone in mm (means ± SD)				
	0 (control)	25 mg/ml	50 mg/ml	100 mg/ml	200 mg/ml
*Candida albicans*					
Day 1	04.52 ± 0.35	04.18 ± 0.21	03.76 ± 0.32	03.49 ± 0.20	03.06 ± 0.34
Day 2	07.68 ± 0.31	06.91 ± 0.40	06.11 ± 0.41	05.01 ± 0.22	04.58 ± 0.44
Day 3	09.18 ± 0.91	08.87 ± 0.27	08.50 ± 0.96	08.01 ± 0.52	07.93 ± 1.03
Day 4	11.61 ± 0.41	11.16 ± 0.34	10.51 ± 0.39	09.65 ± 0.42	09.38 ± 0.37
Day 5	17.45 ± 0.43	16.73 ± 0.38	16.32 ± 0.27	15.53 ± 0.44	16.63 ± 0.49
Day 6	23.53 ± 0.45	22.70 ± 0.42	22.12 ± 0.19	20.53 ± 0.94	20.30 ± 0.36
Day 7	26.44 ± 0.34	25.68 ± 0.47	24.77 ± 0.40	23.98 ± 0.47	23.52 ± 0.37
*Neosartorya fumigata*					
Day 1	05.67 ± 0.52	05.55 ± 0.10	05.53 ± 0.10	05.53 ± 0.21	04.49 ± 0.34
Day 2	20.14 ± 0.78	19.69 ± 0.37	18.88 ± 0.36	18.06 ± 0.52	17.56 ± 0.48
Day 3	40.05 ± 0.12	38.31 ± 1.02	38.16 ± 0.63	36.70 ± 1.12	35.53 ± 0.23
Day 4	47.56 ± 0.55	47.39 ± 0.29	45.74 ± 0.39	44.99 ± 0.44	44.58 ± 0.37
Day 5	60.03 ± 0.45	59.62 ± 0.23	58.53 ± 0.47	57.79 ± 0.41	57.37 ± 0.46
Day 6	74.08 ± 0.66	73.17 ± 0.46	72.46 ± 0.46	71.77 ± 0.36	71.47 ± 0.38
Day 7	78.79 ± 0.36	78.11 ± 0.65	77.86 ± 0.81	76.09 ± 0.44	75.40 ± 0.39
*Neocosmospora keratoplastica*					
Day 1	04.39 ± 0.63	04.25 ± 0.34	04.03 ± 0.43	03.89 ± 0.09	03.64 ± 0.14
Day 2	11.12 ± 0.27	10.64 ± 0.38	10.13 ± 0.56	09.43 ± 0.59	08.75 ± 0.44
Day 3	21.00 ± 0.39	20.40 ± 0.40	19.68 ± 0.50	19.17 ± 0.43	17.74 ± 0.65
Day 4	29.29 ± 0.48	28.00 ± 0.68	21.13 ± 0.26	26.08 ± 0.28	25.02 ± 0.24
Day 5	51.01 ± 0.26	50.01 ± 0.28	49.26 ± 0.29	48.14 ± 0.29	47.00 ± 0.73
Day 6	66.61 ± 0.51	64.65 ± 0.51	63.63 ± 0.47	62.14 ± 0.62	61.44 ± 0.46
Day 7	73.87 ± 0.36	68.47 ± 0.37	67.79 ± 0.37	67.31 ± 1.52	67.08 ± 0.57

### Antiparasitic activity

**Figure 5.**
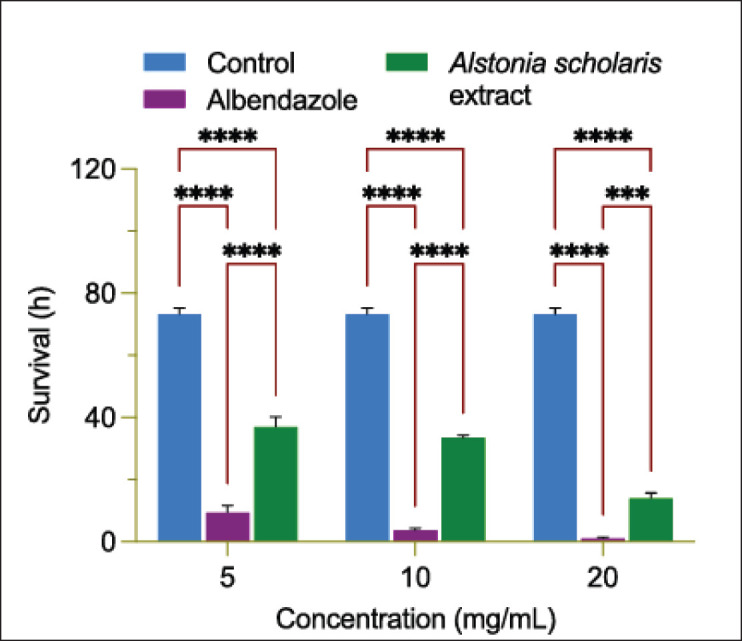
Comparison of the anthelmintic efficacy between *A. scholaris* bark extract and albendazole against *R. echinobothrida*. Values are in means ± standard error of means (*n* = 9); ****p < 0.0001, *** *p* < 0.002.

*Alstonia scholaris* bark extract showed effective concentration-dependent antiparasitic action against the tapeworm, as indicated by the data provided in Table 2. Significant efficacy was observed for all concentrations tested. Statistical group comparison of the negative control and treatment conditions is depicted in Figure 5. The reference antiparasitic drug, albendazole, showed swifter killing activity, but statistical comparison indicated that the plant extract was equally effective (*p* < 0.0001).

**Figure 6.**
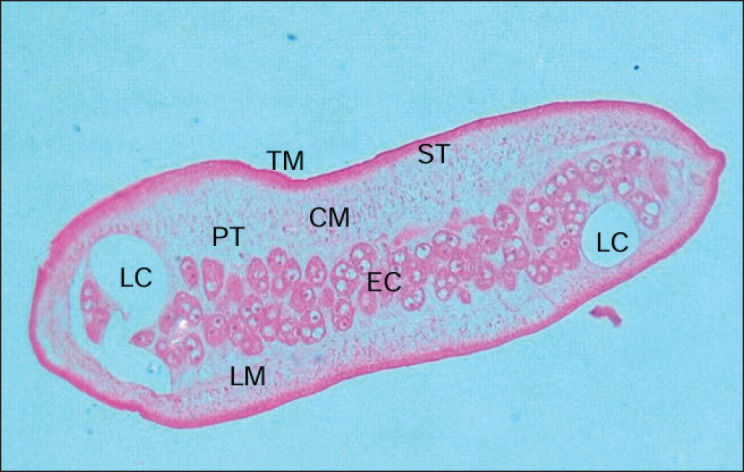
Light micrograph of a histological section of *R. echinobothrida* treated with *A. scholaris* bark extract. The tegument (TM) is the outermost body surface, followed by the sub-tegument (ST) and the longitudinal muscle (LM). Fiber-like red spots are parenchymatous tissue (PT). Circular muscle (CM) surrounds the egg capsules (EC). Lateral canals (LC) are excretory organs.

Light micrographs of the section of tapeworm exposed to *A. scholaris* extract revealed several structural changes (Fig. 6). The most profound effects were seen on the parenchyma tissues that surround the longitudinal muscle and sub-tegumental layers. The otherwise thick parenchyma is reduced to a faint tissue layer, indicating extensive disintegration of the proteinaceous layer. Hazy red spots indicating longitudinal muscle remained. One of the lateral canals is enormously distended with the breakage of the surrounding egg capsule. The circular muscle surrounding the egg capsule was almost entirely disintegrated, represented by thin red filaments.

**Figure 7.**
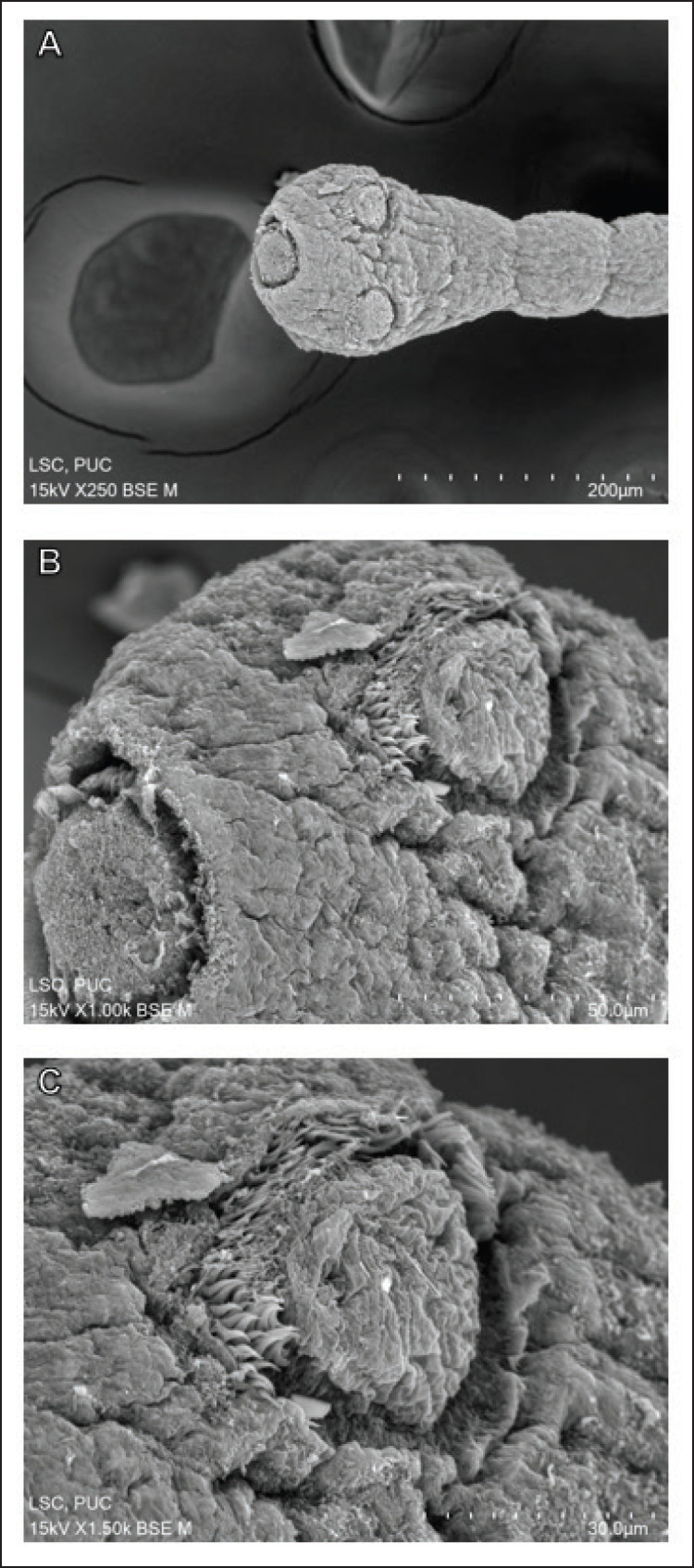
Scanning electron micrographs of the anterior body of *R. echinobothrida* treated with *A. scholaris* bark extract. (A) The scolex and the neck. (B) The scolex is magnified to show the rostellum on the left and one sucker on the top. (C) A sucker showing rows of spines at the top portion.

**Figure 8.**
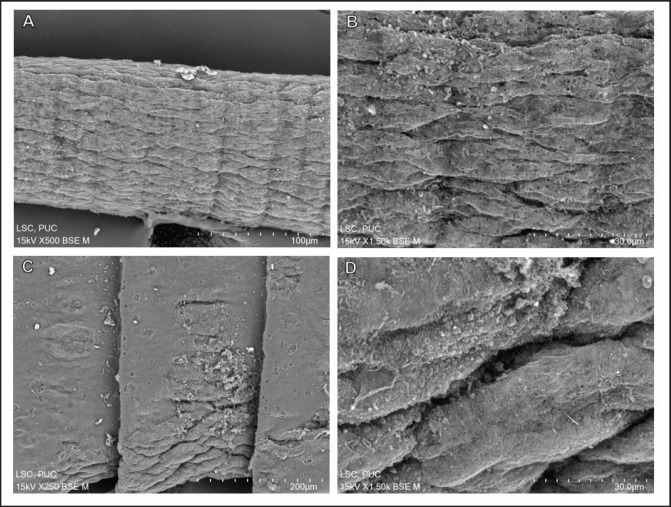
Scanning electron micrographs of the body segments of *R. echinobothrida* treated with *A. scholaris* bark extract. (A) The immature proglottids. (B) Magnification of immature proglottids revealing sharp folds and numerous black pits. (C) The mature proglottids with localized erosions. (G) A magnified mature proglottid.

From scanning electron micrographs, it was evident that the tegumental disruption was extensive throughout the body. The anterior end part called the scolex and the adjoining neck segments showed surface distortion and tegumental shrinkage (Fig. 7A). Critical damage was seen on the eye-like suckers that indicate removal of large chunks of spines (the crucial attachment devices to the host) (Fig. 7A). The tegument on the scolex is extremely corrugated with lumps of abnormal tissue debris attached randomly to the surface (Fig. 7B). Surface peeling was extensive within the rostellum, which lost most of its attachment organs called the hooks. The sucker showed half of the spine still attached, but the other half was removed (Fig. 7C). The immature body segments, or proglottids, are hardly distinguishable from one another due to severe shrinkage and constriction along the longitudinal axis of the body (Fig. 8A). A closer view of the proglottids revealed the sharpness of the tegumental folds, reflecting the severity of the shrinkage. There were clear dark spots indicating pit formation all over the tegument. There were no signs of smooth filaments or microtriches, but instead there was numerous tissue debris adhering to the tegument (Fig. 8B). The mature proglottids showed large portions of their tegument eroded, scarred, and abnormal folds at some points (Fig. 8C). A magnification of the eroded portion of a mature proglottid revealed the formation of lobular blebs, clumping of the microtriches, and formation of pits throughout the tegument (Fig. 8D).

## Discussion

Plant secondary metabolites have been a major focus for lead molecules for drug developments, because of which several pharmaceutical medications have been derived, including those used as antibiotics, anti-cancer, immune suppressant, anti-diabetic, and antiprotozoal drugs [39,40]. Chemical analyses of the Petroleum Ether extract of *A. scholaris* bark in this study established that the species is an abundant source of phytosterols. Different phytosterols, over 250 chemically distinct types identified in different plants, are experimentally demonstrated to have therapeutic potential for debilitating diseases, including cardiovascular diseases, diabetes mellitus, immune disorders, and obesity [41,42]. Health authorities like the European Food Safety Authority and the Nutrition Foundation of Italy had seriously pondered and commended the use of phytosterols as the principle hypolipidemic supplement, as they are specifically effective in reducing low-density lipoprotein of the blood [43]. Evidence in the present data that *A. scholaris* is a valuable source of phytosterols, thus, may attest to many of the plant’s medicinal properties as claimed in traditional medicines.

Another facet of *A. scholaris* found in the present study was its multitudinous antioxidant properties, containing substantial amounts of antioxidant components and having the ability to chemically scavenge cellular oxidants. Free radicals (reactive oxygen species) are the regular by-products, during normal cell metabolisms. However, due to constant cellular activity, they tend to accumulate in the cells beyond the point at which endogenous antioxidants can scavenge them and ultimately turn into highly cytotoxic molecules that are deleterious to the functional and structural biomolecules like DNA, RNA, lipids, and proteins [44]. The oxidative stress produced by such a conglomerate of injurious chemicals becomes the etiological factor of the most dangerous ailments, including cancer, hematological disorders, and pulmonary-cardiac diseases [45]. The extra radical scavengers are obtained from our diet, plants being the primary food sources, thus serving as the major source of exogenous antioxidants [46,47]. We demonstrated that *A. scholaris* does contain a substantial quantity of flavonoids, phenols, and total antioxidants, suggesting that the plant is an invaluable source of nutrients for health benefits. The actual antioxidant activity determined from DPPH and FRAP assays, which are the standard tests for free radical chemical activity [48,49], also justifiably corroborated the overall antioxidant property of the plant.

Species of *Alstonia* are documented to have antifungal activities. The leaf extract of *A. rupestris* showed mild inhibition of plant fungal pathogens such as *Alternaria alternata* and *Phytophthora capsici* [50]. Extracts of *Alstonia macrophylla* leaves showed weak antifungal activity against human dermal pathogens like *Trichophyton rubrum,* *Trichophyton mentagrophytes*, and *Microsporum gypseum* [51]. Venenatine, an indole alkaloid isolated from *A. venenata*, caused 10% inhibition against *Alternaria brassicicola, Fusarium udum, Ustilago cynodontis*, and *Aspergillus flavus* [52]. We demonstrated in this study that *A. scholaris* bark extract was effective against *C. albicans*, *N. fumigata*, and *N. keratoplastica*, which are some of the most impactful pathogenic species in animals and humans. The plant is more efficacious than other *Alstonia* species, particularly against *C. albicans*, in which 10.9% inhibition was observed. The infection of *C. albicans*, known as candidiasis, manifests most commonly in the skin, digestive tract, and vagina, sometimes leading to life-threatening conditions. It becomes a more serious clinical issue because of the emergence of antimicrobial resistance in the fungi [53,54]. *Neosartorya fumigata* is one of the most common airborne infections and deadliest pathogens in immunocompromised individuals [55]. *Neocosmospora keratoplastica* is remarkable in that it causes severe eye infection (keratitis) and is becoming an emerging pathogen with drug resistance [56]. The observation that *A. scholaris* exhibited antifungal activities against these pathogenic fungal species unveils the potential pharmacological value of the plant.

Furthermore, we found that the plant was highly effective against a parasitic tapeworm. Antiparasitic resistance is considered one of the most critical impediments in the animal industry, resulting in massive economic losses [7,56]. The reports of diminished effectiveness of the available drugs and possible development of total drug resistance are medical concerns of grave danger [6,57]. Common anthelmintic drugs like albendazole and praziquantel have been documented to show varying degrees of structural damage to tapeworms, including tegumental distortion, erosion, and removal of microstretches, and loss of spines on the suckers [38,58]. In helminth parasites, drugs enter the body through the general body surface and thus directly attack the body-host interface to induce cellular and structural damage [59,60]. Removal of spines, tegumental erosion, removal of microtriches, and general shrinkage observed in this investigation indicated the strong anthelmintic effects of *A. scholaris*. Thus, while a need for new or improved drugs is pressing, it will be an interesting avenue if *A. scholaris* contains such lead molecules, as suggested by the present findings.

## Conclusion

*Alstonia scholaris* bark extract was demonstrated to possess health benefits as indicated by its antioxidant contents and capacity to scavenge cellular oxidants. It was found to be rich in phytosterols. It showed activity against three important pathogenic fungi: *C. albicans, N. fumigata*, and *N. keratoplastica*. It was most effective against the most pathogenic species, *C. albicans*. It also exhibited antiparasitic activity against the tapeworm, *R. echinobothrida*. Light and scanning microscopy attested to the characteristic antiparasitic action with varying detrimental effects on the parasite. The study validates the plant as a strong antipathogenic agent and warrants deeper exploration into the pharmacological principle and medicinal application.

## List of abbreviations

ATCC, American Type Culture Collection; DHT, dibutylhydroxytoluene; DPPH, 2,2-diphenyl-1-picrylhydrazyl; FRAP, ferric ion-reducing antioxidant power; IC50, half-maximal inhibitory concentration; MDR, multidrug resistance; OD, optical density; PBS, phosphate-buffered saline; SBM, sulfinylbismethane; XDR, extensively drug resistant.
